# Efficient Haze Removal from a Single Image Using a DCP-Based Lightweight U-Net Neural Network Model

**DOI:** 10.3390/s24123746

**Published:** 2024-06-09

**Authors:** Yunho Han, Jiyoung Kim, Jinyoung Lee, Jae-Ho Nah, Yo-Sung Ho, Woo-Chan Park

**Affiliations:** 1 Department of Computer Science and Engineering, Sejong University, Seoul 05006, Republic of Korea; yhhan@rayman.sejong.ac.kr (Y.H.); jykim@rayman.sejong.ac.kr (J.K.); 2 Korea Electronics Technology Institute, Seongnam 13509, Republic of Korea; jylee@keti.re.kr; 3 Department of Computer Science, Sangmyung University, Seoul 03016, Republic of Korea; jaeho.nah@smu.ac.kr; 4 EXARION, Seoul 05006, Republic of Korea; hoyosung@gmail.com

**Keywords:** convolutional neural network, image degradation, dehaze, defog, u-net, dark channel prior

## Abstract

In this paper, we propose a lightweight U-net architecture neural network model based on Dark Channel Prior (DCP) for efficient haze (fog) removal with a single input. The existing DCP requires high computational complexity in its operation. These computations are challenging to accelerate, and the problem is exacerbated when dealing with high-resolution images (videos), making it very difficult to apply to general-purpose applications. Our proposed model addresses this issue by employing a two-stage neural network structure, replacing the computationally complex operations of the conventional DCP with easily accelerated convolution operations to achieve high-quality fog removal. Furthermore, our proposed model is designed with an intuitive structure using a relatively small number of parameters (2M), utilizing resources efficiently. These features demonstrate the effectiveness and efficiency of the proposed model for fog removal. The experimental results show that the proposed neural network model achieves an average Peak Signal-to-Noise Ratio (PSNR) of 26.65 dB and a Structural Similarity Index Measure (SSIM) of 0.88, indicating an improvement in the average PSNR of 11.5 dB and in SSIM of 0.22 compared to the conventional DCP. This shows that the proposed neural network achieves comparable results to CNN-based neural networks that have achieved SOTA-class performance, despite its intuitive structure with a relatively small number of parameters.

## 1. Introduction

With the rapid advancement of artificial intelligence (AI) technology in recent years, there have been significant attempts to utilize it to improve performance in various applications. For example, in areas such as autonomous driving and object detection, AI-based methods have achieved significant performance improvements and have received positive responses from the market. However, the information used as the basis for AI decisions can be corrupted by noise in the acquisition process due to various causes. Such information degradation not only leads to a significant distortion in visual quality but also harms the learning performance of AI technology. Therefore, it is important to address the issue of information degradation due to noise.

Image (video) degradation occurs due to various factors such as camera aging, compression, haze (fog), and inclement weather. Among them, haze is a phenomenon caused by a variety of factors, including suspended particles such as dust and smoke in the atmosphere and the weather. When haze is present, images captured by a camera sensor exhibit a combination of quality degradation, including color distortion, contrast loss, and a loss of depth. Image dehazing [1] is a research field that aims to restore image information corrupted by haze to its pre-damaged state.

In image dehazing research, prior-based methods [2,3,4,5] have been proposed, but more recently, learning methods utilizing artificial neural networks have been explored [6]. As verified in many denoising studies, methods utilizing neural networks perform well in dehazing; however, they suffer from one or more of the following limitations.

First, they require high-performance computing resources. In general, good-performing neural networks consist of complex structures with many parameters [1,6,7]. Denoising, such as dehazing, is often performed in the preprocessing part of the main process, and complex neural network structures can be burdensome for later processes.

Second, they require additional input. Haze is characterized by distortion or a loss of various types of information. Multimodal neural networks use various specialized sensors (LR-Radar, LIDAR, camera, S/M-Radar, ultrasound, etc.) to collect auxiliary information and perform reconstruction based on them [8,9]. This method can improve the quality of the results by making the sensors work in a complementary relationship, but it requires a separate process to reconstruct the data due to the characteristics of different sensor information. For example, using LiDAR and Radar information requires reconstructing them into a single 3D map. These tasks can adversely affect the performance of the entire process.

Third, they are limited in their applicability to general-purpose applications. Neural networks used to solve real-world application scenarios are often complex in structure and require excessive computational costs [10]. This makes it difficult to apply them to low-end, low-power devices such as common cameras and IoT devices, as they cannot handle them. Furthermore, additional specialized sensor inputs may require excessive sensor hardware costs, creating a barrier to their entry into real-world applications.

To address the above issues, we implement a Dark Channel Prior (DCP)-based [2] image dehazing neural network with an intuitive structure that is easy to apply in real-world applications. Our neural network structure is organized in the following way to achieve this goal.

First, we utilize iterations of convolutional operations, which are relatively easy to accelerate, to replace the complex DCP operations. DCP performs several computations that are difficult to accelerate in the process of obtaining the transmission map and then performs high-complexity computations such as soft matting in the process of refining it. Since we replaced these processes with inference using neural networks, we can say that we have improved the relative computational complexity.

Second, it requires only a single RGB image as input. It does not utilize any additional auxiliary inputs for effective dehazing; instead, it only uses a neural network to fill in the missing information. The neural network performs dehazing in two stages, with the first stage inferring the transmission map and adjusting the image contrast to compensate for the missing information. This approach facilitates the application of neural networks in real-world applications without the need for additional auxiliary inputs.

Third, we implement a U-net-structured [11] sub-neural network for effective dehazing. First of all, we make the existing U-net neural network lightweight for robust feature extraction. Then, we apply several proven techniques for quality complementation and efficient training and configure the sub-neural network to specialize in denoising. Finally, we organize the structure of the sub-neural network into blocks to facilitate pipelining. As a result, our neural network consists of relatively few parameters and can efficiently perform high-quality dehazing.

This paper is organized as follows. Section 2 introduces related work and describes the DCP on which this paper is based. Section 3 describes the proposed lightweight U-net-structured neural network specialized for DCP-based haze removal. Section 4 describes the experimental results and analysis, and Section 5 concludes this study and discusses future work.

## 2. Related Works

This section provides an overview of dehazing algorithms and components developed over the past few years. Section 2.1 and Section 2.2 introduce dehazing research before and after applying deep learning, and Section 2.3 introduces DCP.

### 2.1. Traditional Prior-Based Methods

In early dehazing research, several studies were conducted to complement the prior statistical knowledge that haze distorts and loses information in an image. For example, Schechner et al. [3] proposed a method to remove haze by taking pictures with different polarization filters and then using the differences to dehaze them. Similarly, Narasimhan and Nayar [4] proposed a method for obtaining in-depth information from multiple photos taken at the same location on different days (in different climatic conditions). These approaches are limited in their applicability to real-world situations because they do not take a single image as input and do not use generic RGB images. Studies such as Schechner et al. [3] and Narasimhan et al. [4] have many difficulties in obtaining data because they need to obtain multiple images in different conditions at the same place to obtain data.

In addition, several physical models based on prior have been proposed, but the most commonly used techniques to date are Dark Channel Prior (DCP) [2] and Color Attention Prior (CAP) [5]. Collectively, these techniques have high computational complexity and serious limitations in terms of assumptions, resulting in significant color distortion on subjects of certain colors (white and black, respectively).

### 2.2. Deep Learning-Based Methods

As artificial intelligence has shown good performance in various fields, many CNN models have been proposed in the field of image noise processing that easily handle continuous data. CBDNet [12] is a model that aims to remove noise that occurs in the real world and aims to improve the performance of denoising neural network models that are trained to remove Additive White Gaussian Noise (AWGN) but do not perform well with real complex noise.

AOD-Net [13] is characterized by the fact that it is based on a self-reconstructed atmospheric scattering model, unlike most dehazing models [14,15] that focus on the process of estimating transmission maps and atmospheric light based on physical models such as DCP and CAP. DCSC-OPE-Net [16] is a haze removal model aiming to improve visibility and object identification in complex road environments. The neural network is based on MSBDN [17] as the backbone, and it goes through a sub-neural network that performs coarse dehazing by adding color attention and a sub-neural network that extracts detailed features and then uses a DCP transmission map to supplement the details to output the final dehazing image. MSDN-DCP [18] proposes a multi-scale residual neural network with relatively few parameters (3.158 M) to remove haze with low complexity. It is characterized by replacing the convolutional layer of the existing U-net neural network with a FEM (Feature Extraction Module), an FFM (Feature Fusion Module) for feature extraction, and a DCRM (Dark Channel Refinement Module) that utilizes the dark channel value of DCP obtained in advance as a guide image.

FFA-NET [19] presents a block structure that uses channel and pixel attentional techniques successively and implements a neural network that iterates N times. It is characterized by deciding what to focus on at the channel level and where to focus at the pixel level and capturing features by emphasizing them. To remove haze in adverse conditions such as tunnels, Shi et al. [20] constructed a GAN-based double-branching neural network. The neural network is characterized by the parallel operation of a sub-neural network with an encoder–decoder structure to extract low-frequency features and a multi-scale dense residual sub-neural network to supplement the details of the image. Chen et al. [21] proposed a model that is trained to remove haze through unsupervised learning.

TridentNet [22] and TL+CDF [23] are neural networks that achieved good performance in the Image Dehazing section of the NTIRE Challenge. They are implemented in structures that use multiple auxiliary neural networks to extract features and concatenate the results. Each auxiliary neural network specializes in extracting different features and complements each other. As such, common CNN models are often implemented as complex structures that use many parameters to improve performance, or they use multiple inputs.

### 2.3. Dark Channel Prior (DCP)

DCP is a representative physical model based on the statistical knowledge that “in a local area free of haze, except for the sky, pixels have a very low density of at least one of the ‘R’, ‘G’, and ‘B’ channels”. This can be expressed as follows.

(1) $$J^{dark}\left(x\right)=\underset{c\in \left\{r,g,b\right\}}{min}\left(\underset{y\in \Omega \left(x\right)}{min}\left(J^{c}\left(y\right)\right)\right),$$

**Figure 1:**
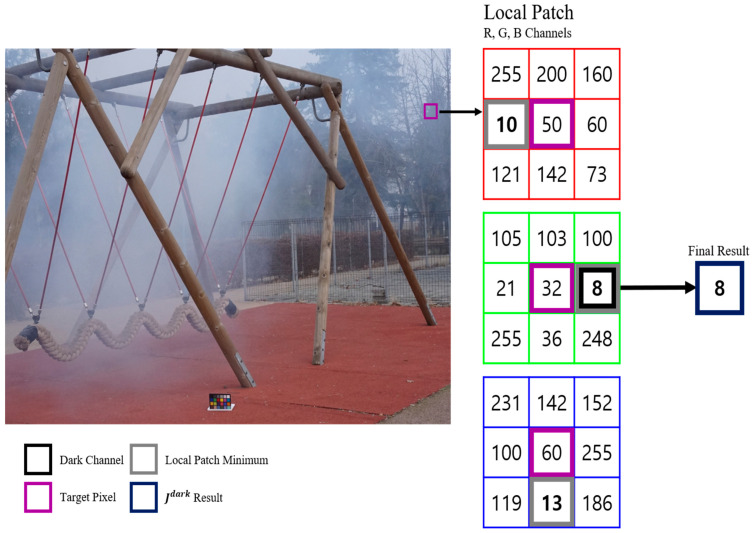
The process of obtaining the dark channel value.

Equation (1) represents the process of formulating the dark channel value in DCP, as illustrated in Figure 1. In this context, $J^{dark}$ denotes the dark channel, $J^{c}$ signifies the channels of the pixel, where x represents a two-dimensional vector indicating the positions of each pixel, and $\Omega \left(x\right)$ denotes the local patch region.

(2) $$I\left(x\right)=J\left(x\right)t\left(x\right)+A\left(1-t\left(x\right)\right),$$

Equation (2) represents the atmospheric scattering model, which is frequently cited to explain atmospheric haze. In this context, $I\left(x\right)$ is the haze image, $J\left(x\right)$ is the atmospheric light, and $t\left(x\right)$ denotes the transmission map. The atmospheric light is determined by selecting the top 0.1% of the dark channel values obtained from Equation (1) to avoid confusion with bright objects in the image and ensure accurate representation of the overall atmospheric light across the entire image.


(3) $$\tilde{t}\left(x\right)=1-\omega \underset{y\in \Omega \left(x\right)}{min}\;\left(\underset{c}{min}\;\frac{I^{c}\left(y\right)}{A^{c}}\right)$$

Applying Equations (1) and (2) allows us to obtain the transmission map $t\left(x\right)$, as expressed in Equation (3). However, due to the nature of atmospheric conditions where days without atmospheric particles are non-existent, ref. [3] introduces a parameter ω (0 < ω ≤ 1) to provide a weight of 0.95 to $t\left(x\right)$. By employing this approach and applying the obtained $t\left(x\right)$ and A to Equation (2), we can calculate $J\left(x\right)$, which represents the dehazed image.

**Figure 2:**
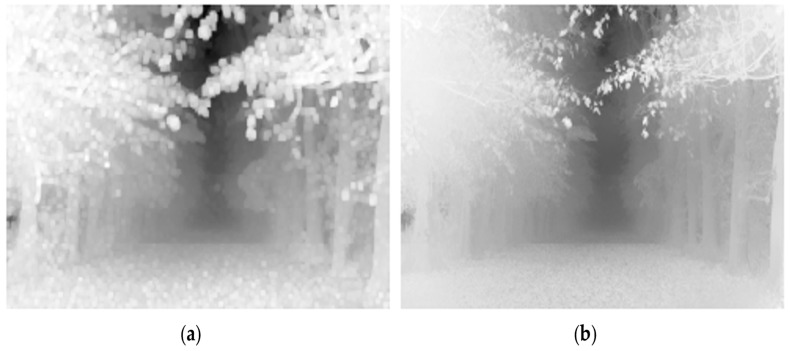
A transmission map obtained via Equation (3) (**a**) and an example of applying the soft matting operation (**b**).

However, $t\left(x\right)$ obtained through the aforementioned method exhibits limitations, showing a blocking effect similar to the left side of Figure 2 due to the use of local patch-based operations. To address this issue, DCP employs soft matting operations and modifies it, as shown on the right side of Figure 2, using it as the transmission map. Nevertheless, soft matting involves computationally intensive iterative optimization processes, and though later replaced with the use of a guided filter, it still performs operations with high complexity [24].

DCP is currently a widely used method, but it exhibits various limitations, prompting several studies to propose solutions. Notably, Shiau et al. [25] address the issue of blurring at the edge of DCP by adaptively calculating the transmission map using a dark channel extracted with 1 × 1 and 15 × 15 size filters. Additionally, Wang et al. [26] propose a human vision-based dark channel calculation method to tackle the problem of over-saturation in the edge area observed in Shiau et al. [25].

## 3. A Proposed Lightweight U-Net-Structured Neural Network Specialized for DCP-Based Dehazing

In this section, we introduce the structure and features of the neural network model for efficient dehazing based on the proposed DCP. To this end, we introduce the structure of the overall neural network model in Section 3.1, describe the structure of the sub-neural networks that comprise the model in Section 3.2, and describe the loss function and training method in Section 3.3.

### 3.1. The Overall Structure of the Proposed Neural Network Model

**Figure 3:**
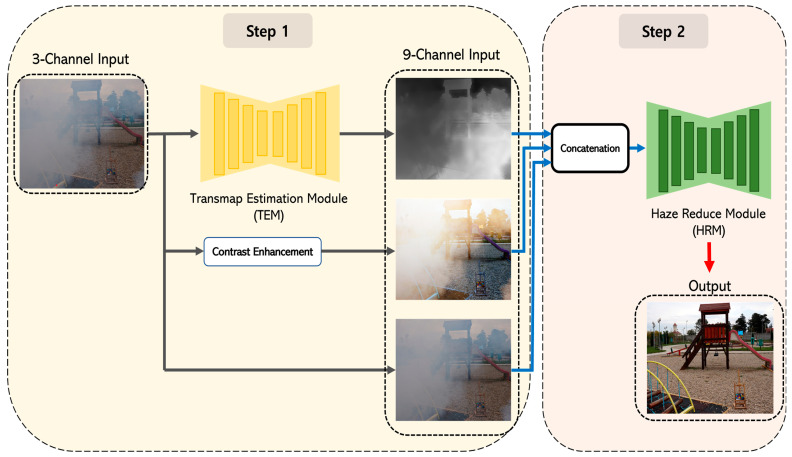
The overall architecture diagram of the proposed neural network model.

Figure 3 shows the overall structure of the proposed dehazing neural network model, which consists of two steps: one to infer the missing information, and two to restore a clean image.

The proposed method uses exclusively RGB images obtained from a regular camera as input. To compensate for the lack of information in the image, we use DCP’s transmission map and contrast enhancement techniques in Step 1. The transmission map represents the percentage of light passing through the atmosphere, so it can capture information such as haze density and light distribution. In this case, the transmission map is inferred from a sub-neural network TEM (Transmission map Estimation Module). The default TEM feature map (transmap) has one channel. However, in our subsequent work, detailed in Section 5, we needed to adjust the input and output channels of TEM and HRM to multiples to facilitate the HW architecture design. To achieve this, we extended the transmap to three channels during the implementation phase. This approach is common in image processing libraries such as OpenCV and PyTorch, and heuristically, it did not significantly affect the results, as the values are not altered during the channel augmentation process [27,28].

To briefly explain how the channels are augmented, we first copy the one-channel feature map to create three layers. These layers are then stacked closely together and reconstructed into a three-channel feature map. The expanded three-channel feature map can be viewed as an RGB image with the same values (grayscale image) copied to each channel (‘R’, ‘G’, ‘B’). This method allows us to expand a one-channel feature map to a three-channel feature map without any distortion or loss of information.

In addition, the contrast-enhanced image can compensate for color, edges, and chromatic contrast lost in the haze. In order to implement this, we used the method based on histogram equalization. This method can effectively improve the contrast by evenly distributing distorted values based on the histogram. The default contrast-enhanced feature map has three channels.

In Step 2, we utilize the information obtained in Step 1 to dehaze the input image. First, we concatenate the output of Step 1 with the initial input image. This is used as input to the second-stage neural network, and the final result is a clean image with the haze removed. The clean image is then inferred by a sub-neural network, the Haze Reduction Module (HRM). The structure of the sub-neural network is described in detail in Section 3.2.

**Figure 4:**
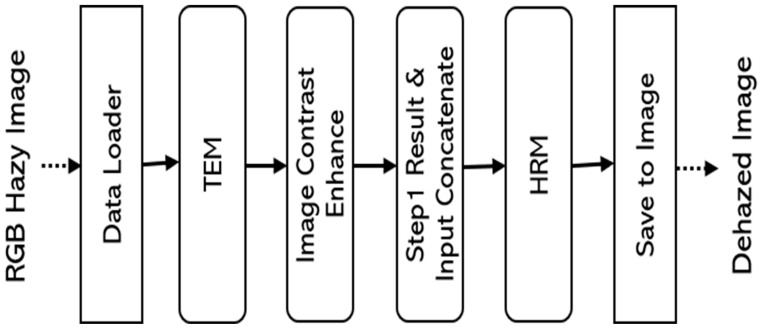
Overall neural network model processing sequence block diagram.

Figure 4 shows a block diagram of the order in which the neural network model is processed. Depending on the implementation, the TEM and image contrast-enhance stages can be processed in parallel. The results of Step 1 are concatenated with the input image and passed to Step 2, the HRM, which finally removes the haze.

### 3.2. Structure and Features of the Sub-Neural Networks That Make up a Neural Network Model

**Figure 5:**
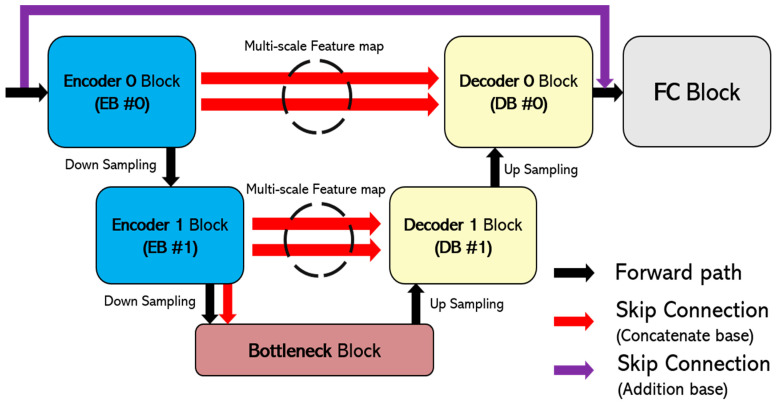
Block diagram of proposed sub-neural network.

Figure 5 shows the structure of the sub-neural network, reconstructed from the U-net neural network. The sub-neural network was constructed to effectively lighten the existing U-net model while maintaining the highest possible quality of the resulting image. To this end, it is configured to specialize in denoising by applying several proven techniques, such as organizing blocks to facilitate multi-scale feature capturing and strengthening connections by adding many skip connections between blocks. The sub-neural network consists of two encoder blocks (EBs), two decoder blocks (DBs), one bottleneck, and one FC block, and performs operations in turn according to the order of the layers within each block.

**Figure 6:**
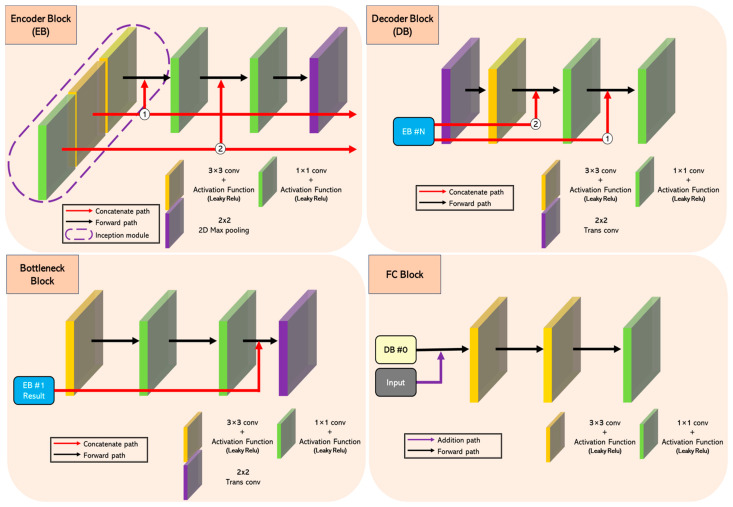
A structural diagram of the EB (encoder block), DB (decoder block), bottleneck block, and FC (fully connected) block constituting the sub-neural network.

The top left of Figure 6 shows the structure of an EB. The EB consists of one pooling layer for down-sampling and five convolutional layers for feature extraction. The convolutional layers are divided into three convolutional layers located in the forward path and two convolutional layers organized in parallel. The two parallel layers play the role of the first layer in the forward path and the inception module [29]. This structure facilitates the capture of multi-scale contexts, enabling complex learning of hierarchical information within blocks. The feature maps generated through the inception module are connected internally to the second and third layers in the form of dense connections [30] and externally to the DB in the form of skip connections (concatenated base) at symmetrical locations.

The bottom left of Figure 6 shows the structure of the bottleneck block. It consists of three convolutional layers in the forward path and one inverse convolutional layer for up-sampling. The resulting feature map from EB 1, passed through the skip connection, is concatenated with the input of the trans-convolution layer.

The top right of Figure 6 shows the structure of the DB. The DB consists of one inverse convolutional layer for up-sampling and three convolutional layers for reconstructing fine-grained location information. The DB is externally connected to the symmetrically located EBs via skip connections. The feature maps passed through the skip connection are concatenated with the inputs of the second and third layers, which are also internally at the corresponding scale because they must be at the same scale to be used with the forward path. For example, the feature map passed through the concatenated path ① of the EB is the result of a 3 × 3 convolution layer and activation function (Leaky ReLU) in the EB. This feature map is then passed internally to the input of the first 1 × 1 convolution layer after the inception module and externally to the input of the last 1 × 1 convolution layer of the DB. This structure complements the detailed information that is easily lost in the forward path and helps to act as a guide to correct some distorted location information during the up-sampling process.

Finally, the bottom right corner of Figure 6 shows the structure of the FC block. The FC layer consists of three convolutional layers, where the input of the first layer is connected to the input of the auxiliary neural network in the form of a residual connection [7]. This structure can help learn low-level features by minimizing the loss of information as it passes through the neural network. The only exception is the convolution layer of the FC block, which does not perform any active function operations.

The TEM and HRM are implemented with the same structure, but the number of channels that make up the first convolutional layer of EB 0 and the last convolutional layer of the FC block are different and use different loss functions. For example, TEM uses a three-channel input in the first layer of EB 0 and a one-channel output in the last layer of the FC block. However, the HRM uses nine-channel inputs in the EB 0’s first layer and three-channel outputs in the FC block’s last layer.

### 3.3. Loss Functions and Training Strategies

**Figure 7:**
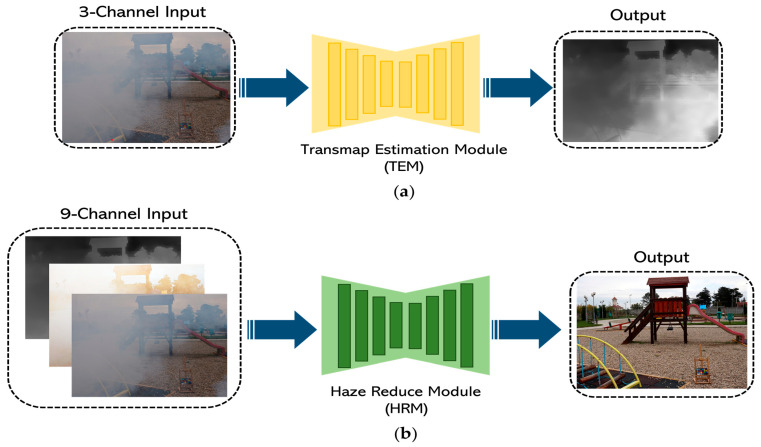
Examples of sub-neural network training. (**a**) Example of TEM training. (**b**) Example of HRM training.

The TEM and HRM perform training independently to ensure efficient learning and better overall neural network performance. Figure 7 shows an example of training in action. Once trained, they operate as a single neural network model with the hazy image as input and a clean, dehazed image as ground truth (GT), as shown in Figure 3.


(4) $$L\left({TEM}_{i}(x),GT_{i}(x)\right)=\frac{1}{m}\sum_{i=1}^{m}\;{∥{TEM}_{i}(x)-GT_{i}(x)∥}^{2}$$

The TEM uses a typical RGB three-channel image with haze as input to infer the transmission map. For inference, the transmission map of the input image extracted through DCP is utilized as GT for training. As a loss function, we use MSE loss as shown in Equation (4) to infer a predicted feature map that is close to the correct transmission map.


(5) $$f(n)=\left\{\begin{array}{ll} L\left(DM_{i}(x),GT_{i}(x)\right)=\frac{1}{m}\sum_{i=1}^{m}\;{∥DM_{i}(x)-GT_{i}(x)∥}^{2}, & \mathrm{if}\;\mathrm{Epoch}\;<10\\ L^{SSIM}(DM)=1-SSIM(\tilde{DM}), & \mathrm{if}\;\mathrm{Epoch}\;\geq 10 \end{array}\right.$$

The HRM concatenates the resulting feature maps from Step 1 and the original input image as input. For inference, a clean image with haze removed is utilized as GT for training. As a loss function, MSE loss is used up to 10 epochs as shown in Equation (5), and SSIM loss is used for subsequent learning. Using SSIM loss alone instead of this approach will not significantly impede learning, but using MSE loss initially will allow the results to converge quickly and then allow for detailed feature learning. This approach is indirect, but it allowed us to learn effectively [31].

## 4. Experiments and Results

In this chapter, we present our experimental setup and results. First, we describe the experimental environment and the datasets used in Section 4.1, followed by the experimental results of the full neural network model compared to traditional DCP in Section 4.2. Finally, in Section 4.3, we perform additional comparisons with existing dehazing neural networks for a more diverse comparison.

### 4.1. Experimental Environment and Datasets

The neural network model was implemented using the Python 3.9.16 version language and the PyTorch library on an Intel^®^ Core™ i7-8700k 3.7GHz CPU with Samsung 64GB RAM. To accelerate the training process, an Nvidia RTX 3080 GPU was utilized.

**Figure 8:**
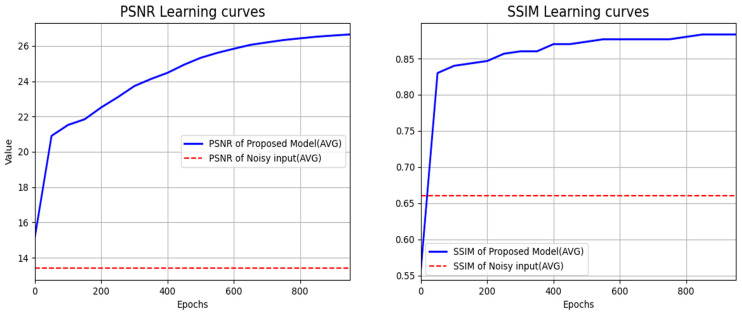
PSNR and SSIM learning curves for the proposed neural network (2M version). The blue lines represent the trends of metric changes as training progresses, while the red dashed lines indicate the PSNR and SSIM metrics for the noisy images used as input.

The RESIDE-β, RESIDE-standard, and NH-HAZE datasets were used as benchmarks for the validation and performance evaluation of the neural network model [32,33]. For accurate evaluation, the resolution of the datasets was uniformly changed to 1280 × 720. In addition to the proposed 2M version, we also ran a 1M version in order to check the difference in the number of parameters that make up the sub-neural network. Each version has different parameters for composing the sub-neural networks. To be more specific, our final proposed 2M version consists of 1M parameters each for TEM and HRM, while the 1M version uses half that number. The neural network optimizer was Adam, the learning rate was set to 0.0005, and a gamma of 0.96 was applied to the learning rate every 10 learning steps using the step scheduler. The training was performed in 1000 epochs, and Figure 8 shows the PSNR and SSIM values measured every 50 epochs during the actual training process in the form of learning curves.

Table 1 shows the main components of datasets that we used as benchmarks. RESIDE datasets [32] consist of large datasets comprising synthetic and real-world hazy images. In addition to the Standard version, RESIDE provides various extended versions with concepts such as V0, β version, and more. For our experiments, we did not use the entire dataset but extracted the Indoor Training Set (ITS) from the RESIDE-Standard dataset and the Outdoor Training Set (OTS) from the RESIDE-β dataset. In particular, the ITS provides hazy data with 10 levels of noisiness (13,990 by default), and we only used the haze with the highest noisiness level. Before augmentation, the base resolution is 620 × 460.

NH-HAZE datasets [33] are specifically designed for image haze removal. Creating synthetic haze is challenging due to its inhomogeneous nature. NH-HAZE overcomes this challenge by using a nebulizer to implement non-homogeneous physical features like real-world haze. However, since NH-HAZE is a relatively small dataset consisting of 55 photos, the amount of data for training is absolutely insufficient. In order to compensate for this, we first categorized 40 of the 55 images as the training set, 5 as the validation set, and the remaining 10 as the test set. Then, we used simple transformations such as random rotation (90, 180, 270 degrees), horizontal flip, vertical flip, and random cropping to create a relatively large number of images. The base resolution for NH-HAZE datasets is 1600 × 1200.

### 4.2. Experimental Results and Performance Comparison with DCP

**Figure 9:**
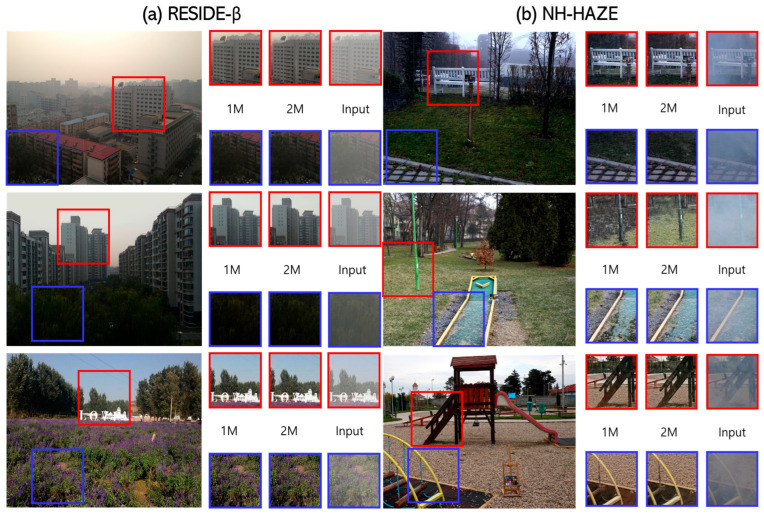
Ground truth (GT) and resulting images of HRM. (**a**) RESIDE-β(OTS) datasets. (**b**) NH-HAZE datasets.

Figure 9 shows the resulting images of the 1M and 2M versions of the full neural network model, and Table 2 shows the performance evaluation results. PSNR, a representative image quality quantitative evaluation metric, was used for performance evaluation. The numbers in Table 2 refer to the PSNR values of the experimental results of the input, 1M, and 2M versions of the neural network, and the experimental results of the existing DCP [2] compared to GT. First, as shown in Table 2, both the 1M and 2M versions have significantly improved performance compared to the existing DCP method. However, we can see that the 1M version has slight performance degradation due to detailed context and color distortion compared to the 2M version.

**Figure 10:**
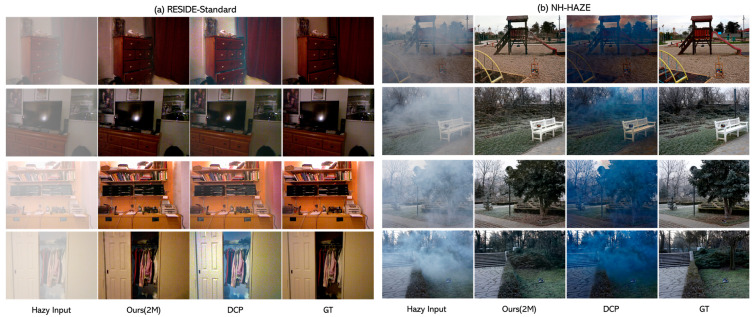
A comparison of the experimental results between the proposed neural network model and DCP. (**a**) The experimental results on the Indoor Training Set (ITS) of RESIDE datasets. (**b**) The experimental results on NH-HAZE datasets.

Figure 10 shows the input image and the results of the 2M version of the experiment and provides a visual comparison of the GT image with the traditional DCP results. While DCP suffers from frequent color distortion and halo effects due to the limitations of its underlying assumptions, our neural network shows a relatively good recovery of image details.

**Figure 11:**
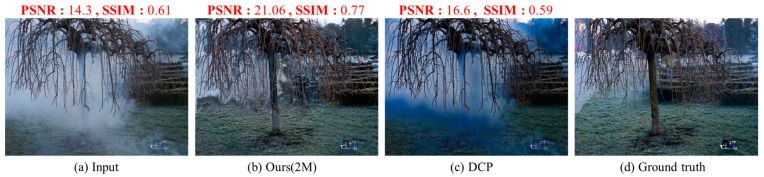
The experimental result images for the second scene of NH-HAZE. (**a**) The input image, (**b**) the experimental result for the 2M version of the neural network model, (**c**) the experimental result for conventional DCP, and (**d**) ground truth (GT).

Figure 11 shows the experimental result images and performance evaluation results for the second scene of the NH-HAZE dataset. For a dramatic comparison, we use the scene in which DCP increases PSNR but decreases SSIM.

In Figure 11c, the results from DCP show severe color distortion and halo effects in the haze area. The existing DCP performs additional operations such as soft matting and filtering to reduce the block effect of the transmission map, which is one of the causes of this, but it does not solve it completely. This problem is also reflected in the performance evaluation results, which show an improvement in PSNR but a decrease in SSIM. This color distortion is a characteristic of most of the DCP-enhanced haze results and occurs throughout the image.

In Figure 11b, the experimental results of the proposed neural network 2M parameter model show relatively good dehazing, but there still remains a slight halo effect. This is likely because the DCP-based transmission map from the TEM was trained on the target and then used as input to the HRM. This problem can be easily solved by adding a series of operations before passing the results to the HRM, which will be discussed later in Section 5.

### 4.3. Experimental Results and Performance Comparison with Other Neural Networks

Table 3 compares the proposed neural network (2M) in terms of the number of parameters and performance evaluation results with the methods suggested in [2,13,19,22,23]. RESIDE-Standard (ITS) and NH-HAZE datasets were used as benchmarks for an objective comparison. Exceptionally, [2] is not based on deep learning, so the number of parameters are not specified. The underlined values in each column indicate the highest performance metric. Refs. [13,19] are used for comparison as widely adopted models in the majority of dehazing studies. Refs. [22,23] are models that achieved outstanding performance in the NTIRE 2020 Dehazing Challenge, with [22] in particular achieving state-of-the-art (SOTA) performance.

The performance evaluation metrics for each dataset in Table 3 exhibit significant differences overall. This can be attributed to the fact that NH-HAZE datasets are created to reflect the non-homogeneous nature of real haze, unlike RESIDE-Standard (ITS). Assuming severe haze conditions in real-life scenarios, the performance evaluation results on NH-HAZE datasets can be considered closer to practical usability.

First, according to the performance evaluation results of RESIDE-Standard (ITS) datasets in Table 3, the neural networks proposed in [19,22,23] achieved a better performance than the neural network implemented in this paper. However, ref. [18] shows significant performance degradation compared to our neural network on the NH-HAZE dataset. This means that our proposed neural network can perform better than [19] in situations where it needs to remove irregular noise, such as haze in the real world. Refs. [22,23] are implemented with a structure that uses multiple auxiliary neural networks, each capturing different features. This structure requires many parameters, and in fact, they used 23 and 25 times more parameters than our neural network, respectively. Nevertheless, we can see that our proposed neural network achieves a comparable performance to [22] and a better performance than [23] on the NH-HAZE dataset. In addition, Table 3 shows that our proposed neural network achieves the highest SSIM in the performance evaluation performed on the NH-HAZE dataset. These results collectively show that our proposed neural network is very efficient in removing real-world haze.

Because Table 3 shows mixed results, we would like to analyze them. In RESIDE-Standard (ITS), recent neural network approaches [19,22,23] show higher PSNR and SSIM values than ours. However, they commonly use more complex networks than ours; refs. [22,23] used 23 and 25 times more parameters than our neural networks, respectively. In NH-HAZE datasets, ref. [19] shows a significantly worse performance than ours, and our network’s results are comparable to [22] and better than [23]. In particular, our model achieves the highest SSIM value among the models. In both datasets, our model achieves significantly better results than another lightweight model [13]. According to these results, we can conclude that our approach is not only lightweight but also particularly suitable to removing non-homogeneous haze.

## 5. Conclusions and Future Work

In this paper, we propose a DCP-based lightweight U-net-structured neural network model that performs efficient denoising with a single input. We propose a two-stage neural network model to perform high-quality dehazing with a single input. In addition, we propose a sub-neural network structure to the existing U-net model that is effectively lightweight and specialized for denoising while maintaining the resulting image quality as much as possible.

As a result of functional verification and performance evaluation through benchmark tests of the neural network, we achieve an average PSNR of 21.63 and an SSIM of 0.76 on the NH-HAZE dataset, which simulates the physical characteristics of real haze. Although it did not achieve SOTA-class performance in another RESIDE-Standard (ITS) dataset, it should be further evaluated for its comparable high-efficiency performance using fewer parameters.

In future work, we plan to apply some of the attentional techniques of the Transformer model to the HRM to improve the quality of the proposed model. Further, lightweight modification is planned for the TEM, which did not show a significant difference between the 1M and 2M versions, and the entire neural network model will be changed to an end-to-end structure to enable fully parallel accelerated processing.

In this paper, we only focused on the structure and implementation of the neural network model. Our future goal is to design a dedicated hardware architecture to enable the proposed neural network model to operate in real time. This requires further research, including simplifying the neural network, designing dedicated buffers, and configuring an efficient memory system. We have designed a nine-channel neural network HW that can accelerate the TEM and HRM, and we are following up by running it in iterative mode. Our ultimate goal is to create a highly efficient, high-quality, real-time dehazing/denoising system that can be applied to a wide range of applications, from low-power devices such as cameras and IoT devices to applications that require real-time processing such as autonomous driving.

## Figures and Tables

**Table 1:** Components of datasets used as benchmarks.

	RESIDE-Standard (ITS) [32]	RESIDE-β (OTS)	NH-HAZE [33]
Hazy	1399	72,135	55
Clear	1399	72,135	55
Resolution	620 × 460		1600 × 1200

**Table 2:** Performance (PSNR in dB) evaluation results for the overall neural network model.

Datasets	Input	Ours (1M)	Ours (2M)	DCP [2]
NH-Haze	11.47	20.84	21.63	12.58
RESIDE-β (OTS)	15.61	28.83	30.72	15.76
RESIDE-Standard (ITS)	13.04	26.2	27.6	17.11

**Table 3:** Performance evaluation results and comparison with previous studies.

Methods	Parameters (M)	RESIDE-Standard (ITS)		NH-HAZE	
		PSNR (dB)	SSIM	PSNR (dB)	SSIM
DCP [2]	FILTER	17.11	0.72	12.58	0.45
AOD-NET [13]	0.17	19.06	0.85	13.44	0.413
FFA-NET [19]	4	36.39	0.988	18.51	0.637
TridentNet [22]	46	34.59	0.975	23.06	0.755
TL+CDF [23]	49+1	37.61	0.991	21.44	0.704
Ours (2M)	2	30.72	0.97	21.63	0.76

## Data Availability

Data are available upon request to the corresponding author..
